# Neuroprotective Potential of *Guiera senegalensis* (Combretaceae) Leaf Hydroethanolic Extract against Cholinergic System Dysfunctions and Oxidative Stress in Scopolamine-Induced Cognitive Impairment in Zebrafish (*Danio rerio*)

**DOI:** 10.3390/plants11091149

**Published:** 2022-04-24

**Authors:** Jorelle Linda Kamda Damo, Razvan Stefan Boiangiu, Ion Brinza, Léa Blondelle Kenko Djoumessi, Roland Nhouma Rebe, Balbine Nkwingwa Kamleu, Simon Désiré Nyayi Guedang, Guillaume Woumitna Camdi, Parfait Bouvourné, Eglantine Wado Keugong, Hervé Hervé Abaïssou Ngatanko, Oana Cioanca, Monica Hancianu, Harquin Simplice Foyet, Lucian Hritcu

**Affiliations:** 1 Department of Biological Sciences, Faculty of Science, University of Maroua, Maroua P.O. Box 814, Cameroon; lindajorelle@yahoo.fr (J.L.K.D.); kenkolea@gmail.com (L.B.K.D.); rnhouma@yahoo.com (R.N.R.); kamleubalbine@yahoo.com (B.N.K.); g.nyayi@gmail.com (S.D.N.G.); gcamdi77@gmail.com (G.W.C.); parfaitoel@gmail.com (P.B.); eglantinewado@gmail.com (E.W.K.); abaissouherve@gmail.com (H.H.A.N.); 2 Department of Biology, Faculty of Biology, Alexandru Ioan Cuza University of Iasi, 700506 Iasi, Romania; razvan.boiangiu@uaic.ro (R.S.B.); ion.brinza@student.uaic.ro (I.B.); 3 Department of Pharmacognosy, Faculty of Pharmacy, “Grigore T. Popa” University of Medicine and Pharmacy, 700115 Iasi, Romania; oana.cioanca@umfiasi.ro (O.C.); monica.hamciamu@umfiasi.ro (M.H.)

**Keywords:** *Guiera senegalensis*, scopolamine, Alzheimer’s disease, memory, acetylcholinesterase

## Abstract

*Guiera senegalensis* JF Gmel. (Combretaceae) (GS) is a plant used in traditional medicine in West Africa for the treatment of several diseases, such as epilepsy and depression. However, its potential benefits in improving scopolamine (Sco)-induced memory impairment and brain oxidative stress in zebrafish have been investigated. In the present study, zebrafish (*Danio rerio*) were treated with GS (1, 4, and 8 μg/L) for 19 days as well as Sco (100 µM) 30 min before behavioral tests. Behavioral performance was assessed by the Y-maze test and novel object recognition test (NOR), whereas anxiety response was evaluated in the novel tank diving test (NTT). Subsequently, high-performance liquid chromatography (HPLC) was used to evaluate the GS chemical composition. Sco promoted oxidative stress and acetylcholinesterase (AChE) activity. Moreover, both oxidative stress parameters and AChE activity were ameliorated by GS treatment. Accordingly, the present findings further provided the potential use of GS as a natural, alternative treatment against cognitive disorders associated to Alzheimer’s disease (AD).

## 1. Introduction

Alzheimer’s disease (AD) is a progressive neurodegenerative disorder that causes substantial cognitive and behavioral abnormalities, such as learning difficulties and memory loss [1,2]. Furthermore, neuropsychiatric symptoms such as anxiety and depression are strongly correlated to the pathology [3]. Thus, AD slowly deteriorates all aspects of human living, resulting in incapacitation. The pathogenesis of AD is attributed to senile plaque, neurofibrillary tangle formation, oxidative stress, inflammatory process, and cholinergic dysfunction [4,5].

The cholinergic system is important for memory processing, and the loss of the cholinergic neurons and subsequent reduction results in learning and memory dysfunction that are typical of AD [6]. Acetylcholinesterase inhibitors (tacrine, rivastigmine, donepezil and galantamine) are the most commonly prescribed medications for AD. They provide clinical relief while also improving cognitive function. Additionally, they improve cholinergic neurotransmission by boosting acetylcholine availability in cholinergic synapses [7,8]. However, adverse cholinergic side effects, limited effectiveness, hepatotoxicity, or poor bioavailability are some limitations of these drugs [9,10]. As a result, a variety of plants have been utilized to treat neurodegenerative disorders and could be attractive candidates for the development of safe, effective, and multitargeted drugs for the treatment of AD [2].

Scopolamine (Sco) is a nonselective muscarinic cholinergic antagonist, commonly used as a model for the study of AD [2,8,9]. It causes cognitive impairment by lowering the acetylcholine levels in the brain and boosting AChE activity [11]. Moreover, Sco also causes cellular changes such as antioxidative defense system, increased oxidative stress, mitochondrial malfunction, apoptosis and neuroinflammation. It promotes the deposition of amyloid peptide and the hyperphosphorylation of Tau proteins [12]. Several investigations on neurodegenerative diseases have been carried out using zebrafish (*Danio rerio*) as an animal model due to the homology of several genes between humans and zebrafish [13,14]. Sco has been demonstrated to impair learning and memory in zebrafish, indicating that it could be used as a model for the research of AD. As a result, zebrafish are a useful transitional model between in vitro receptor and cell-based assays and traditional mammalian drug screening models [15,16]. In West Africa, *Guiera senegalensis* (Combretaceae) is used for the treatment of headaches, epilepsy, depression, mental disorders, insomnia, hallucinations, loss of consciousness, sinusitis, abdominal pain, malaria, diarrhea, and vomiting [17,18]. Phytochemically, *G. senegalensis* leaves contain many compounds, including tannins, flavonoids, alkaloids, triterpenes, coumarins and saponosides [18,19]. Previous in vitro studies have revealed anticholinesterase, antioxidant, and anti-inflammatory properties of this plant [20,21,22,23].

In this study, we investigated whether *G. senegalensis* leaf hydroethanolic extract prevents Sco-induced cognitive dysfunction in zebrafish.

## 2. Results and Discussion

### 2.1. Chemical Composition of Guiera senegalensis Extract

**Figure 1:**
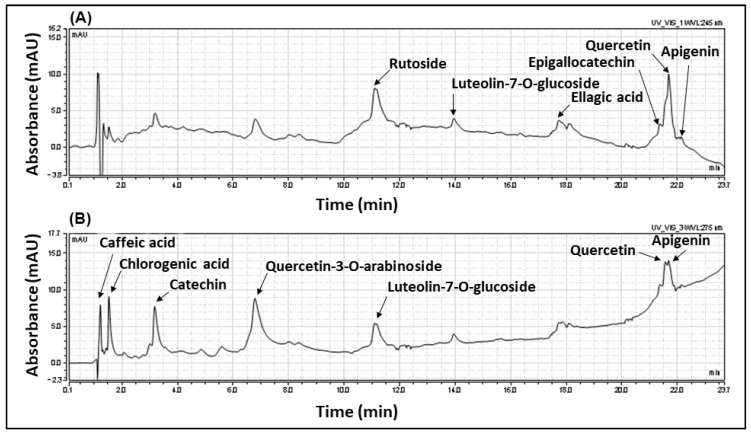
HPLC-PAD chromatography profile at (**A**) 245 nm and (**B**) 275 nm for the flavonoids and polyphenolic acids of the *Guiera senegalensis* leaf hydroethanolic extract.

GS extract was analyzed by High-Performance Liquid Chromatography coupled with a Photodiode Array Detector (HPLC-PAD) to determine its chemical profile. The results indicated the presence of several flavonoids and polyphenolic acids such as quercetin-3-O-arabinoside, catechin, luteolin-7-O-glucoside, rutoside, quercetin, apigenin, epigallocatechin, caffeic acid, chlorogenic acid, cinnamic acid, and ellagic acid (Figure 1). The amounts detected are: quercetin-3-O-arabinoside—316.30 µg/g dry extract, catechin—108.57 µg/g dry extract, luteolin-7-O-glucoside—54.46 µg/g dry extract, rutoside—205.71 µg/g dry extract, quercetin—306.86 µg/g dry extract, apigenin—141.65 µg/g dry extract, epigallocatechin—120.05 µg/g dry extract, caffeic acid—126.45 µg/g dry extract, chlorogenic acid—55.20 µg/g dry extract, cinnamic acid—106.14 µg/g dry extract and ellagic acid—80.13 µg/g dry extract (Table 1).

Various authors have demonstrated the presence of more than 46 compounds in the vegetal material from *G. senegalensis* that is used in traditional medicine [24,25,26]. According to other published data, similar compounds to our results were identified in the leaves of *G. senegalensis*; however, most of the published data contains a general identification without the quantifications of these compounds. The extractability is correlated with the plant material, the solvent, and the preparation process. Flavonoids and gallic acid derivatives were most often reported along with different polyphenolic acids. The chemical composition indicates that the investigated extract is rich in polyphenols (flavonoids, catechins and polyphenolic acids) that are recognized as natural antioxidants. The free hydroxyl moieties allow interaction with cell membranes and induce a protective mechanism against free radicals. Moreover, quercetin, apigenin, and catechin derivatives act as scavengers against reactive oxygen species and inhibit oxidative enzymes such as lipoxygenases, preventing cell denaturation [24,25].

### 2.2. Effects on Anxious Behavior and Spatial Memory in Zebrafish in NTT, Y-Maze and NOR Tests

**Figure 2:**
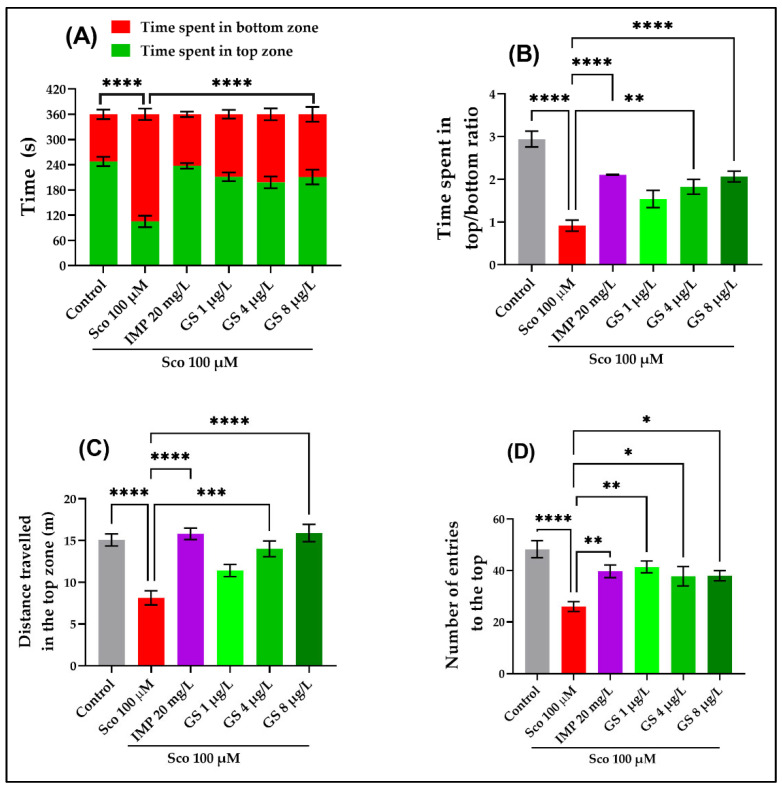
NTT results for *Guiera senegalensis* hydroethanolic extract (GS: 1 µg/L, 4 µg/L and 8 µg/L). (**A**) Time spent in the top/bottom zone. (**B**) Time spent in top/bottom ratio. (**C**) Total distance travelled in the top zone. (**D**) Number of entries to the top zone. Data are expressed as mean ± S.E.M. (n = 10). For Tukey’s post hoc analyses: * *p* < 0.01, ** *p* < 0.001, *** *p* < 0.0001 and **** *p* < 0.00001.

The novel tank diving test (NTT) has been frequently used to study anxiety related reaction in zebrafish. The one-way ANOVA revealed a significant effect of GS treatment on the time spent in top of the tank (*p* < 0.0001) (Figure 2A), on the time spent in top/bottom ratio (*p* < 0.0001) (Figure 2B), on the total distance traveled in the top zone (*p* < 0.0001) (Figure 2C), and on the number of entries to the top (*p* < 0.01; *p* < 0.001) (Figure 2D). The time spent in the top zone of the tank (*p* < 0.0001) (Figure 2A), the time spent in top/bottom ratio (*p* < 0.0001) (Figure 2B), the distance traveled in the top zone (*p* < 0.0001) (Figure 2C), and the number of entries to the top (*p* < 0.0001) (Figure 2D) were significantly reduced by Sco administration as compared to the control group. Moreover, the Sco-administration decreased locomotory pattern as compared to the control group. In contrast, the anxiolytic-like effect of GS treatment at all doses (1 µg/L, 4 µg/L and 8 µg/L) was noticed in the Sco zebrafish. Imipramine (IMP), used as a positive reference drug, exhibited anxiolytic effects.

**Figure 3:**
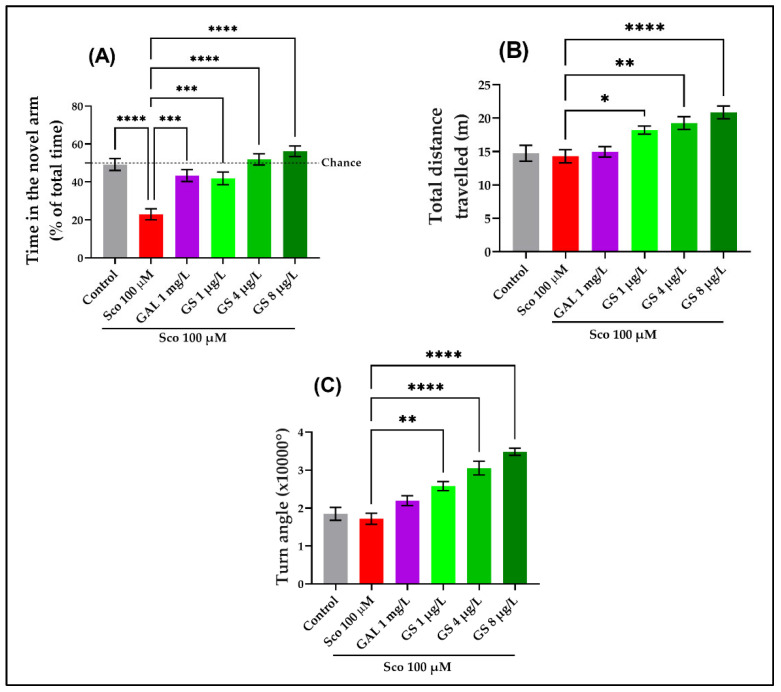
*Guiera senegalensis* hydroethanolic extract (GS: 1 µg/L, 4 µg/L and 8 µg/L) improved locomotion and memory in the Y-maze test. (**A**) Time spent in the novel arm. (**B**) Total distance traveled. (**C**) Turn angle. Results are expressed as mean ± S.E.M. (n = 10). For Tukey’s post hoc analyses: * *p* < 0.01, ** *p* < 0.001, *** *p* < 0.0001 and **** *p* < 0.00001.

The one-way ANOVA revealed a significant effect of GS treatment on the time spent in the novel arm (*p* < 0.00001) (Figure 3A), on the total distance traveled (*p* < 0.00001) (Figure 3B), and on the turn angle (*p* < 0.00001) (Figure 3C) in the Y-maze test. Additionally, the Sco treatment induced spatial memory deficit, as evidenced by a significant decrease in the time spent in the novel arm (*p* < 0.00001) (Figure 3A). Moreover, the administration of Sco induced hypolocomotion, as evidenced by the decrease in the total distance traveled (Figure 3B) and the turn angle (Figure 3C) as compared to the control group. The GS treatment significantly reduced the hypolocomotion and memory problems that the Sco administration had caused. Galantamine (GAL), which was utilized as a positive control, had memory-enhancing benefits.

**Figure 4:**
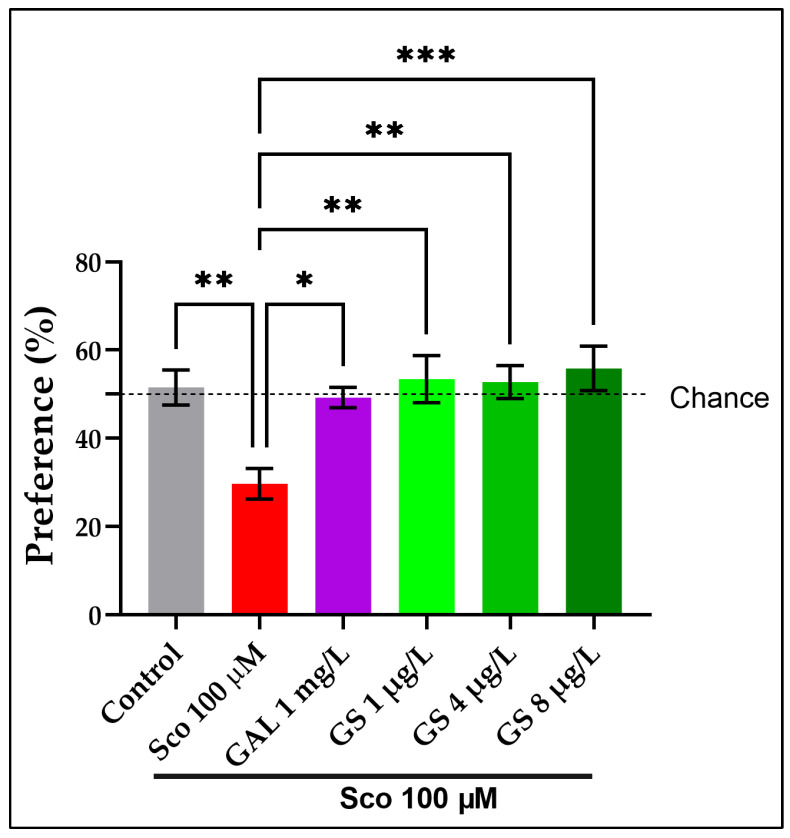
*Guiera senegalensis* hydroethanolic extract (GS: 1 µg/L, 4 µg/L and 8 µg/L) improved memory in the novel object recognition (NOR) test. The figure represents the percentages of preference in different groups. Results are expressed as mean ± S.E.M. (n = 10). For Tukey’s post hoc analyses: * *p* < 0.01, ** *p* < 0.001, and *** *p* < 0.0001.

Furthermore, a NOR test was also employed to assess the effects of GS on memory (Figure 4). The one-way ANOVA revealed a significant effect of GS treatment on the preference percentages (*p* < 0.0001). The NOR test showed a significant reduction in the percentages of preference (*p* < 0.001) (Figure 4) in Sco-induced zebrafish as compared with the control group. The GS-treated groups (1 µg/L, 4 µg/L and 8 µg/L) showed a tendency to increase the preference percentages. The behavioral data revealed that GAL, when taken as a positive reference drug, exhibited memory-enhancing effects.

Our findings are consistent with those of previous researchers, indicating that GS has a neuroprotective effect. Sombie et al. [27] suggested that the galls from *G. senegalensis* could be used as a new potential source of natural neuroprotective and antioxidant components. Additionally, Kinda et al. [17] demonstrated that *G. senegalensis* is used for psychiatric and neuropsychiatric disorder treatment in the Hauts-Bassins region of Burkina Faso. The authors attributed the observed effects to the presence of the neuroprotective compounds. GS could be considered as a therapeutic medication with a high potential to improve cognitive impairments based on these outcomes. In fact, we used the Sco zebrafish model to show that GS possesses cognitive-enhancing and anxiolytic characteristics.

### 2.3. Effects on the Brain AChE Activity

**Figure 5:**
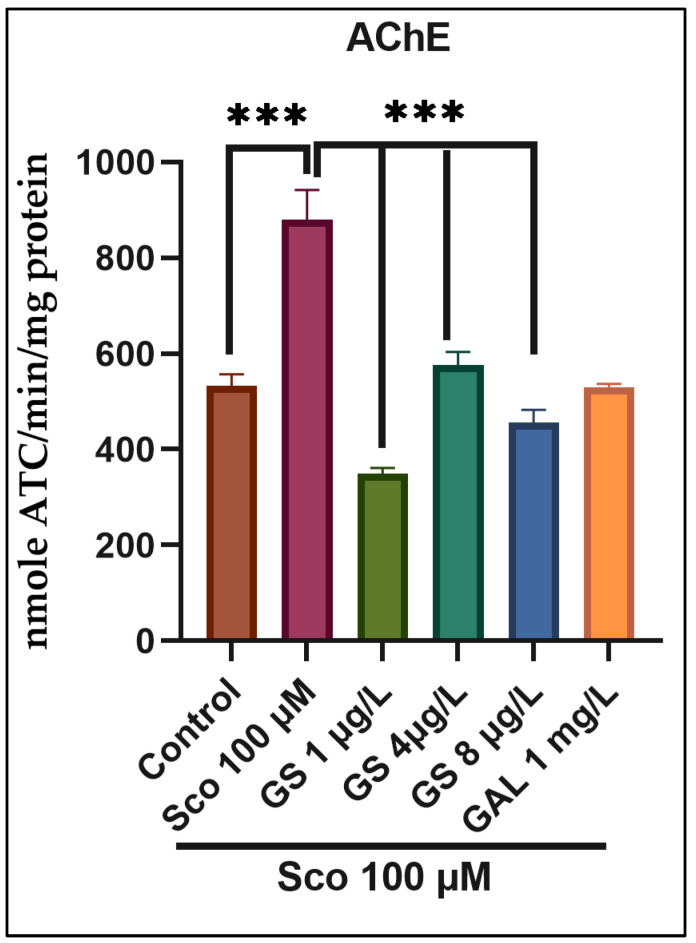
Effects of *Guiera senegalensis* hydroethanolic extract (GS: 1 µg/L, 4 µg/L and 8 µg/L) on Sco-induced increasing in the activity of the acetylcholinesterase (AChE). Each column represents mean ± S.E.M. of 10 zebrafish. For Tukey’s post hoc analyses: *** *p* < 0.0001.

To further investigate the mechanism of GS in the Sco zebrafish model, AChE activity was analyzed. AChE activity represented cholinergic status in mild cognitive impairment [28]. The cholinergic system plays a vital role in memory processing, whereby cholinergic neurons are lost. AChE decreases acetylcholine levels and their subsequent decrease results in the learning and memory dysfunction characteristics of AD [6]. Furthermore, AD therapy implies AChE inhibitors and thereby enhances cholinergic neurotransmission [2,8]. The zebrafish treated with Sco significantly increased AChE activity compared with the control group (*p* < 0.0001) (Figure 5). Moreover, the GS treatment significantly decreased at all doses the Sco-mediated AChE exacerbation (*p* < 0.0001) (Figure 5). In the NTT, Y-maze, and NOR tests, GS demonstrated cholinesterase inhibitory potential, which correlates with improved memory characteristics in zebrafish. AChE inhibition is a therapeutic target for the treatment of dementia such as AD, senile dementia, and Parkinson’s disease dementia. The AChE rise generated by Sco in the zebrafish brain was greatly reduced by GS, especially at low doses. Hippocampal AChE is a vital component for dementia-related memory deficits and the underlying mechanism of cognitive disorders because it modulates cognitive performance [6] Because AChE inhibition has been shown to be an effective treatment for dementia and related cognitive problems [29,30], this inhibition by GS confirms its anti-amnesia capabilities.

### 2.4. Effects on Brain Antioxidant Capacity

**Figure 6:**
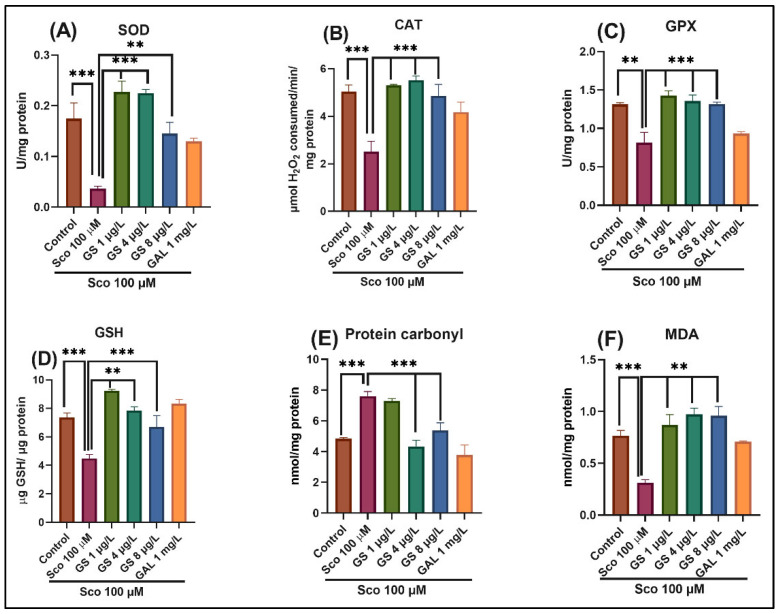
*Guiera senegalensis* hydroethanolic extract (GS: 1 µg/L, 4 µg/L and 8 µg/L) improved brain antioxidant status. (**A**–**C**) Representation of the enzymes’ specific activity: superoxide dismutase (SOD), catalase (CAT), and glutathione peroxidase (GPX) in different groups; (**D**–**F**) Representation of the reduced glutathione (GSH), protein carbonyl, and malondialdehyde (MDA) levels in different groups. Values are means ± S.E.M. (*n* = 10). For Tukey’s post hoc analyses: ** *p* < 0.001 and *** *p* < 0.0001.

To further investigate whether GS increases antioxidant capacity in vivo, the oxidative stress markers were evaluated. Exposure to Sco resulted in significant reduction in the specific activities of the antioxidant enzymes superoxide dismutase (SOD) (*p* < 0.0001) (Figure 6A), catalase (CAT) (*p* < 0.0001) (Figure 6B), glutathione peroxidase (GPX) (*p* < 0.001) (Figure 6C), the total content of reduced glutathione (GSH) (*p* < 0.001) (Figure 6D), along with increased levels of protein carbonyl (*p* < 0.0001) (Figure 6E) and lipid peroxidation (malondialdehyde—MDA) (*p* < 0.0001) (Figure 6F) when compared with the control group. Furthermore, GS treatment (1 µg/L, 4 µg/L and 8 µg/L) significantly prevented the decrease in SOD (*p* < 0.001) (Figure 6A), CAT specific activity (*p* < 0.0001) (Figure 6B), GPX (*p* < 0.0001) (Figure 6C) specific activities and total content of reduced GSH (*p* < 0.0001) (Figure 6D). Moreover, GS treatment reduced protein and lipid peroxidation by significantly decreasing protein carbonyl levels (*p* < 0.0001) (Figure 6E) and MDA levels (*p* < 0.001) (Figure 6F) in Sco-treated animals.

Sco-induced amnesia has been linked to an increase in oxidative stress in brain tissue [31,32]. In accordance with this, the present study demonstrated that Sco significantly decreased the antioxidant enzyme (SOD, CAT, and GPX) activities and the reduced GSH content and increased the MDA and protein carbonyl levels in the zebrafish brain. Meanwhile, the administration of GS significantly enhanced antioxidant defense by suppressing the increase of MDA and protein carbonyl and the decrease of GSH level in the brain of zebrafish treated with Sco. Oxidative stress plays an important role in AD pathogenesis due to pro-oxidant compound accumulation [33]. In fact, oxidative stress is caused by an imbalance between oxidant and antioxidant systems. An accumulation of reactive oxygen species (ROS) causes damage to lipids, cellular proteins, and nucleic acid, and leads consequences such as lipid peroxidation, protein oxidation, and DNA oxidation For protection against oxidative cell damage, antioxidant enzymes such as SOD, GPX, and CAT are included in the defense system [34]. MDA is a lipid peroxidation reaction product that serves as an oxidative stress marker. The level of MDA in tissues can indicate the degree of lipid peroxidation. [2]. SOD is an important enzyme in the fight against ROS-induced tissue damage. It catalyzes the transfer of the superoxide anion to hydrogen peroxide, and then catalase transforms hydrogen peroxide into water [35,36]. GSH is an endogenous antioxidant, which can react directly with ROS or act with GPX to decrease hydrogen peroxide and lipid peroxide levels in tissues [5,37]. Antioxidant therapy has therefore been suggested for the prevention and treatment of AD [2,38]. Indeed, numerous studies have linked Sco-induced memory loss to a rise in brain oxidative stress [2,8,9]. Our data suggested that GS exhibits neuroprotective effects against oxidative stress. The current observations are corroborated by research on *Guiera senegalensis* antioxidant effects in vitro due to its phenolic and flavonoid components [22,23].

**Figure 7:**
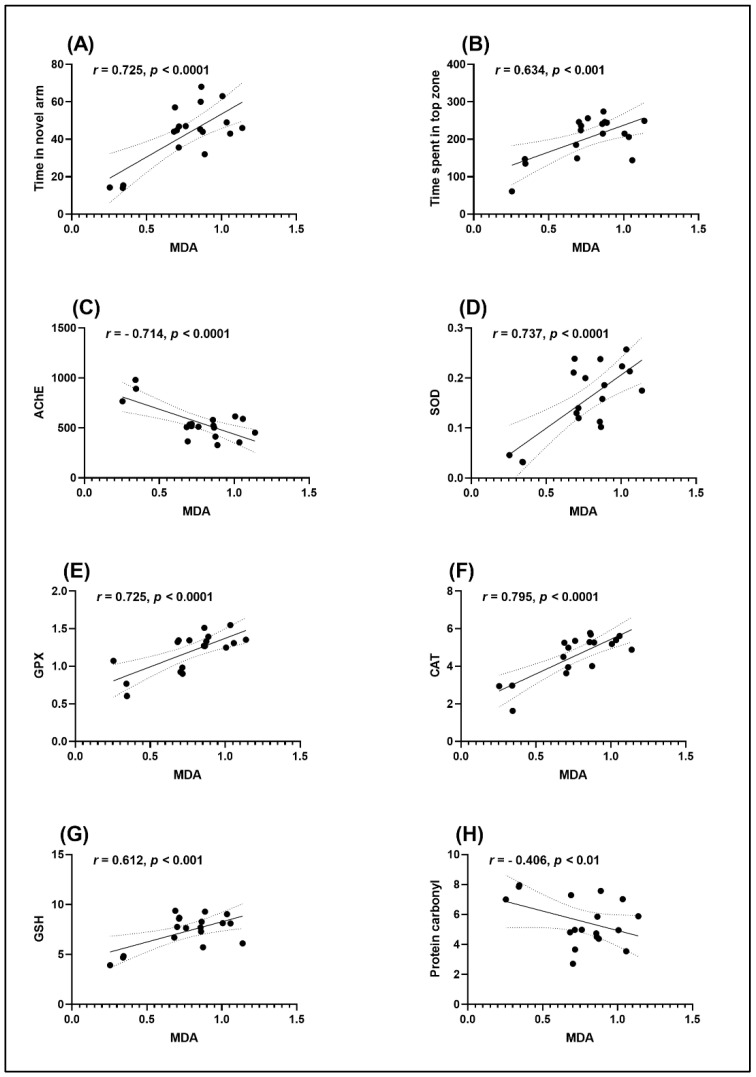
Correlation analyses between behavioral and biochemical parameters (Pearson’s correlation). Data expressed are time in the novel arm (s), time spent in the top zone (s), AChE (nmol/min/mg protein), SOD (U/mg protein), GPX (U/mg protein), CAT (U/mg protein), GSH (µg GSH/µg protein), protein carbonyl (nmol/mg protein) and MDA (nmol/mg protein). (**A**) Time in novel arm vs. MDA (*n* = 10, *r* = 0.725, *p* < 0.0001); (**B**) Time spent in the tope zone vs. MDA (*n* = 10, *r* = 0.634, *p* < 0.001); (**C**) AChE vs. MDA (*n* = 10, *r* = −0.714, *p* < 0.0001); (**D**) SOD vs. MDA (*n* = 10, *r* = 0.737, *p* < 0.0001); (**E**) GPX vs. MDA (*n* = 10, *r* = 0.725, *p* < 0.0001), (**F**) CAT vs. MDA (*n* = 10, *r* = 0.795, *p* < 0.0001), (**G**) GSH vs. MDA (*n* = 10, *r* = 0.612, *p* < 0.001) and (**H**) Protein carbonyl vs. MDA (*n* = 10, *r* = −0.406, *p* < 0.01).

Pearson correlation coefficient (*r*) was calculated to assess the relationship between cognition performance, antioxidant enzymes, and lipid peroxidation (Figure 7). Results revealed significant positive correlation between time in the novel arm vs. MDA (*n* =10, *r* = 0.725, *p* < 0.0001) (Figure 7A), time spent in the top zone vs. MDA (*n* =10, *r* = 0.634, *p* < 0.001) (Figure 7B), SOD vs. MDA (*n* =10, *r* = 0.737, *p* < 0.0001) (Figure 7D), GPX vs. MDA (*n* =10, *r* = 0.725, *p* < 0.0001) (Figure 7E), CAT vs. MDA (*n* =10, *r* = 0.795, *p* < 0.0001) (Figure 7F), and GSH vs. MDA (*n* =10, *r* = 0.612, *p* < 0.001) (Figure 7G). Significant negative correlations between AChE vs. MDA (*n* =10, *r* = −0.714, *p* < 0.0001) (Figure 7C), and protein carbonyl vs. MDA (*n* =10, *r* = −0.406, *p* < 0.01) (Figure 7H) were noticed.

Parvez et al. [39] reported a strong correlation between antioxidant and antiviral potential of *G. senegalensis* leaf extract. Using the Pearson correlation coefficient (*r*) approach, we found that better memory function in Sco-treated rats is linked to an increase in antioxidant enzyme activity, as well as a decreased level of lipid peroxidation and AChE activity, indicating that GS has a neuroprotective profile.

## 3. Materials and Methods

### 3.1. Plant Material and Extraction

Fresh leaves of *Guiera senegalensis* were harvested in June 2020 in the locality of Touloum (Far North, Cameroon). The botanical identification of the plant was carried out at the National Herbarium of Cameroon compared to a botanical sample of Sabatié B. No. 699, which has been found and registered under No. 49837/HNC.

The extraction protocol was performed as previously described by Imam et al. [40]. Fresh leaves of *G. senegalensis* were dried in the shade for a week, then reduced to a fine powder. Three hundred grams (300 g) of the resulting powder was macerated for 72 h in a solvent consisting of 80% ethanol and 20% distilled water. The mixture was filtered using a Whatman paper. The result was evaporated in a rotavapor at 80 °C to remove the ethanol, then in oven at 50 °C for 48 h to remove water. The hydroethanolic extraction yield was 7.67% (*v*/*v*).

### 3.2. High-Performance Liquid Chromatograph (HPLC-PDA)

The Ultra-Performance Liquid Chromatography (UPLC) coupled with a photodiode array detector (PDA) was used for the GS analysis. Initially, the extract was diluted in a methanol–water mixture (ratio 3:7) to obtain a stock solution of a concentration of 4 mg/mL. The solution was clarified with a 0.2 µm Millipore filter. Volumes of 5 µL were injected in the LC-PDA Thermo UltiMate 3000 (Pro Analysis Systems, Bucharest, Romania) system equipped with a Luna C18 column (Phenomenex, CA, USA) (150 × 4.6 mm, 100 Å). The mobile phase consisting of an A (acetonitrile with 1% phosphoric acid)/B (aqueous solution of 1% phosphoric acid) mixture was obtained automatically during analysis by the quaternary pump and the initial and final flow were 1 mL/min. During sample analysis, the flow rate was established at 0.8 mL/min. Simultaneous detection was performed for a total elution time of 25 min at four wavelengths: 245 nm (polyphenols), 275 nm (flavonoids), 330 nm (polyphenolic acids), and 520 nm (anthocyanins). Aliquots of the diluted extract were injected in a linearity gradient. Standard stock solutions (catechin, epicatechin, caffeic acid, ellagic acid, p-coumaric acid, cinnamic acid, chlorogenic acid, ferulic acid, luteolin, apigenin, kaempferol, epigallocatechin, quercetin, rutoside, quercetin-3-O-arabinoside, apigenin-7-O-glucoside, luteolin-7-O-glucoside, and ecdysone) were used for calibration curves with a correlation coefficient above 0.9989. Values of 245 ng/mL and 182 ng/mL were calculated as the limit of detection (LOD) and the limit of quantification (LOQ) for rutin and p-coumaric acid, respectively. UV spectra comparison and retention time were used for the identification taking into consideration a match index above 950/1000. All standards and solvents were of HPLC quality and were purchased from Sigma Aldrich (Darmstadt, Germany).

### 3.3. Animals and Treatment

**Figure 8:**
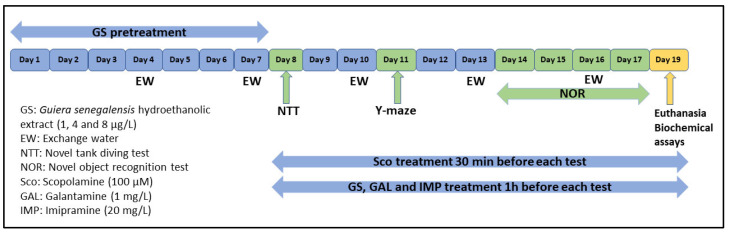
Experimental design procedure for drug administration behavioral study (NTT, NOR, and Y-maze test) and biochemical assays.

A total of 70 adult male and female short-finned *Danio rerio* zebrafish (n = 10 per group) were obtained from Pet Product S.R.L. (Bucharest, Romania). Fish were kept at 26 ± 1 °C on a 14:10-h light/dark cycles in 24 L housing tanks (30 × 30 × 30 cm), provided with constant filtration and aeration (pH = 7.5, dissolved oxygen at 7.20 mg/L, ammonium concentration <0.004 ppm, and a conductivity of 500 μS). Adult fish were fed twice a day with commercial flake fish food (Norwin Norvitall, Norwin, Gadstrup, Denmark). Fish were acclimated for one week before the experiment began. All animals were divided into the following groups: the control group, the scopolamine group (Sco, 100 μM, Sigma–Aldrich, Darmstadt, Germany), three *Guiera senegalensis* treatment groups (GS: 1, 4, and 8 μg/L), the imipramine group (IMP, 20 mg/L, Sigma–Aldrich, Darmstadt, Germany, as a positive control within a novel tank diving test (NTT)), and the galantamine group (GAL, 1 mg/L, Sigma–Aldrich, Darmstadt, Germany, as a positive control within Y-maze and novel object recognition (NOR) tests). The doses of Sco, IMP, and GAL were chosen following a previous report [16]. GS (1, 4, and 8 μg/L) was administered by immersion once daily into a 500 mL glass for 1 h, as well as Sco (100 μM) 30 min before the beginning of behavioral tests [10]. All procedures and protocols (Figure 8) involving the care and use of laboratory animals were approved by the Ethics Committee on Animal Research of the Alexandru Ioan Cuza University of Iași, Romania, Faculty of Biology (No. 15309/30.06.2019) and fully complied with the Directive 2010/63/EU of the European Parliament and of the Council of 22 September 2010 on the protection of animals. Full efforts were made to diminish the use of animals and to improve their wellbeing.

### 3.4. Behavioral Analyses

For the behavioral analysis, a Logitech HD Webcam C922 Pro Stream camera (Logitech, Lausanne, Switzerland) recorded zebrafish behavior, and the videos were analyzed using ANY-maze^®^ software, version 6.3 (Stoelting CO, Wood Dale, IL, USA).

#### 3.4.1. Novel Tank Diving Test (NTT)

The NTT is a specific test used to evaluate both locomotor activity and anxious response in zebrafish as described by Cachat et al. [41]. The NTT consisted of a 1.5 L trapezoidal tank (15.2 × 27.9 × 7.1 cm) divided by a virtual horizontal line into top and bottom sections. Zebrafish were individually assessed for 6 min. The endpoints for locomotor activity consisted of distance traveled (m) and average velocity (m/s). Anxiety-like behavior was determined by quantification of the number of entries to the top, time spent in top (s), average entry duration (s), total distance traveled in the top (m) and freezing duration (s) endpoints.

#### 3.4.2. Y-Maze Test

The Y-maze task was employed for assessing the zebrafish response towards a novel place [42]. The zebrafish position into the novel arm of the Y-shaped glass tank (25 × 8 × 15 cm for each arm, 5 L) was considered an index of memory [43]. The Y-maze arms were assigned as the “start” arm and the “other” arm (always open) and the “novel” arm (blocked in training session and opened in test session). During the training session (5 min), the fish were individually placed in the start arm and the novel arm was closed. After 1 h, the test session (5 min) began, and the fish were again placed in the start arm, but this time, the novel arm was opened. For the locomotor activity, the measured endpoints were total distance traveled (m) and turn angle (°), while the response to novelty was measured by assessing the time spent in novel arm (% of total arm time).

#### 3.4.3. Novel Object Recognition Test (NOR)

The NOR is a commonly used behavioral assay for investigating memory performance in zebrafish [44]. Briefly, zebrafish were subjected to 5 min of acclimation to the novel tank (30 × 30 × 30 cm filled with 6 cm of water) for 3 consecutive days in the absence of the objects. On the fourth day, zebrafish were exposed for 10 min to two identical objects (training phase). One hour after the training phase, one of two identical objects (familiar objects, FO) was randomly replaced with a novel object (NO) and the interaction was monitored for 10 min (testing phase). The preference percentages were calculated as follows: [time of exploration of NO/time of exploration of FO + time of exploration of NO × 100].

### 3.5. Biochemical Parameter Assay

Zebrafish were euthanized by the rapid cooling method [10] (immersion in ice-cold water at 2–4 °C for 10 min) after behavioral testing. The brains were precisely dissected for biochemical analyses. For this, the brains were homogenized in ice 0.1 M potassium phosphate buffer (pH 7.4), 1.15% KCl with Mikro-Dismembrator U mill (Sartorius, New York, NY, USA) equipped with 3 mm diameter magnetic balls (Sartorius Stedim Biotech GmbH, Goettingen, Germany). Samples were centrifuged at 960× *g* for 15 min and the supernatant was used for the estimation of acetylcholinesterase (AChE), superoxide dismutase (SOD), catalase (CAT), glutathione peroxidase (GPX) specific activities, reduced glutathione (GSH), protein carbonyl and malondialdehyde (MDA) levels, following the methods described in detail by Boiangiu et al. [45].

#### 3.5.1. Determination of the AChE Activity

The acetylcholinesterase (AChE) activity was assessed using Ellman’s method [46]. The final volume of the reaction mixture (600 µL) contained 0.26 M phosphate buffer with pH 7.4, 1 mM 5.5′-dithio-bis-2 nitrobenzoic acid (DTNB), and 5 mM acetylthiocholine chloride (ATC). The AChE was measured at 412 nm and the enzyme activity was expressed as nmol of ACT/min per/mg of protein.

#### 3.5.2. Determination of the SOD Activity

The superoxide dismutase (SOD) activity was measured according to the method described by Winterbourn et al. [47]. Each 1.5 mL reaction mixture contained 100 mM TRIS/HCl (pH 7.8), 75 mM NBT, 2 µM riboflavin, 6 mM EDTA, and 200 µL supernatant. Following the blue formazan production, the absorption increases at 560 nm, which is monitored. One unit of SOD is defined as the quantity required to inhibit the rate of NBT reduction by 50%. The enzyme activity was expressed in units/mg protein.

#### 3.5.3. Determination of the CAT Activity

The catalase (CAT) activity in the brain supernatant was determined by the method described by Sinha [48]. The reaction mixture included 150 µL phosphate buffer (0.01 M, pH 7.0) and 100 µL supernatant. 250 µL H_2_O_2_ 0.16 M was added to the start of the reaction, which was then incubated at 37 °C for 1 min before being stopped with 1 mL of dichromate acetic acid reagent. The tubes were placed in a boiling water bath for 15 min, and the green color created during the reaction was measured at 570 nm. In addition, control tubes that were devoid of the enzyme, were treated in parallel. The activity of the enzyme is measured as µmol of H_2_O_2_ consumed/min/mg protein.

#### 3.5.4. Determination of the GPX Activity

Sharma and Gupta [49] described a method for determining the glutathione peroxidase (GPX) activity: 1 mL 0.4 mM phosphate buffer (pH 7.0) containing 0.4 mM EDTA, 1 mL of 5 mM NaN_3_, 1 mL of 4 mM glutathione (GSH), and 200 µL of supernatant were pre-incubated for 5 min at 37 °C. Then, 1 mL of 4 mM H_2_O_2_ was added and incubated at 37 °C for another 5 min. The 5,5′-dithiobis-2-nitrobenzoic acid (DTNB technique was used to determine the GSH excess). One unit of GPX is expressed as the quantity of enzyme required to oxidize for 1 nmol GSH/min. The enzyme activity was measured in units/mg protein.

#### 3.5.5. Determination of the GSH Level

The reduced glutathione (GSH) level in the zebrafish brain supernatant was determined using the Fukuzawa and Tokumura method [50]. After mixing 200 µL of brain supernatant with 1.1 mL of 0.25 M sodium phosphate buffer (pH = 7.4), 130 µL DTNB 0.04% was added. Finally, the mixture was diluted to 1.5 mL with distilled water, and the absorbance was measured at 412 nm. The data were expressed as µg GSH/µg protein.

#### 3.5.6. Determination of the Protein Carbonyl Level

Using a method developed by Oliver et al. [51] and modified by Luo and Wehr [52], the extent of protein oxidation in the brain was determined by detecting the concentration of protein carbonyl groups. The supernatant fraction was divided into two equal aliquots, each with approximately 2 mg of protein. Trichloroacetic acid 10% (TCA, *w*/*v*, final concentration) was used to precipitated both aliquots. Another sample was treated with 2 N HCl at an equivalent volume, while another was treated with 0.2% (*w*/*v*) DNPH in 2 N HCl. Both samples were incubated at 25 °C for 5 min before being mixed. The outcomes were expressed in nmol/mg protein.

#### 3.5.7. Determination of the MDA Level

The MDA end product of lipid peroxidation in brain homogenate was measured quantitatively using the method published by Ohkawa et al. [53]. 200 µL of supernatant was applied and briefly mixed in 0.1 M HCl with 1 mL of 50% trichloroacetic acid in 0.1 M HCl and 1 mL of 26 mM thiobarbituric acid. After vortex mixing, samples were kept at 95 °C for 20 min. After centrifuging the samples for 10 min at 960× *g*, the supernatants were measured at 532 nm. As mentioned, the results were provided as nmol/mg protein.

### 3.6. Statistical Analysis

Results were expressed as mean ± standard error of the mean (S.E.M). Differences between means were analyzed utilizing one-way analysis of variance (ANOVA) followed by Tukey post hoc multiple comparison test, considering treatment as a factor. Statistical significance was set at *p* < 0.05. Statistical analyses were performed by GraphPad Prism 9.0 (GraphPad Software, Inc., San Diego, CA, USA). Correlation between the behavioral scores, enzymatic activities, and lipid peroxidation was estimated by the Pearson correlation coefficient (*r*).

## 4. Conclusions

This study aimed to evaluate the neuroprotective effect of *Guiera senegalensis* leaf hydroethanolic extract by investigating its cognitive-enhancing, anti-AChE, and antioxidant activities in a scopolamine zebrafish model. Thus, the anti-AChE and antioxidant characteristics of GS in the nervous system could explain its cognitive-protecting activities against Sco-induced memory loss. These findings support the use of GS as a natural alternative treatment for cognitive disorders associated with AD.

## Figures and Tables

**Table 1:** Active constituents identified in *Guiera senegalensis* extract sample.

Phytochemical Group	*Guiera senegalensis* Extract	
	Compounds	Quantity (µg/g Dry Extract)
**Flavonoids**	Quercetin-3-O-arabinoside	316.30
	Catechin	108.57
	Luteolin-7-O-glucoside	56.46
	Rutoside	205.71
	Quercetin	306.86
	Apigenin	141.65
	Epigallocatechin	120.05
**Polyphenolic acids**	Caffeic acid	126.45
	Chlorogenic acid	55.20
	Cinnamic acid	106.14
	Ellagic acid	80.13

## Data Availability

The data presented in this study are available on request from the corresponding author.
